# Cross-modal fusion of cytomorphology and ^18^F-FDG PET/CT for non-invasive bone marrow immune microenvironment decoding in multiple myeloma

**DOI:** 10.3389/fimmu.2026.1856163

**Published:** 2026-06-24

**Authors:** Minjie Gao, Guifang Ouyang, Yangguang Liu, Shikun Chen, Kaihong Xu

**Affiliations:** 1 Department of Hematology, The First Affiliated Hospital of Ningbo University, Ningbo, Zhejiang, China; 2 College of Artificial Intelligence, Ningbo University of Finance and Economics, Ningbo, Zhejiang, China

**Keywords:** ^18^F-FDG PET/CT, bone marrow immune microenvironment, computational immunophenotyping, deep learning, hematology, immunotherapy response, multiple myeloma, tumor immunology

## Abstract

**Introduction:**

The bone marrow immune microenvironment (BMME) shapes treatment response in multiple myeloma (MM), yet routine diagnostic workup primarily assesses tumor burden rather than immune competence. We developed ImmunoCast-MM, a cross-modal deep learning framework that extracts immunologically relevant signals from two examinations routinely performed at diagnosis: Wright–Giemsa-stained bone marrow aspirate smears and whole-body ^18^F-FDG PET/CT.

**Methods:**

The cytomorphology branch used DinoBloom embeddings to classify individual cells across five hierarchical levels. The PET/CT branch generated a multi-organ inflammation fingerprint from tumor, spleen, lymph node, and diffuse bone marrow compartments. A contrastive fusion module aligned the two imaging modalities with a flow cytometry reference panel and generated an Immune Dysfunction Index (IDI) along a learned effector–suppressor axis. ImmunoCast-MM was evaluated retrospectively in 243 patients with newly diagnosed MM. Associations with flow cytometric measurements, progression-free survival, and daratumumab response were assessed, with adjustment for International Staging System stage, cytogenetic risk, and age.

**Results:**

On a held-out validation subset, decoded cytomorphologic indices correlated with matched flow cytometric fractions, with Spearman *ρ* values of 0.68–0.81 across three decoded index–panel pairs; all Benjamini–Hochberg-adjusted (p) values were (<0.001). Unsupervised clustering of the fused embeddings identified immune-competent and immune-exhausted archetypes that differed in progression-free survival and response to daratumumab. Separately, adding the IDI to conventional risk markers increased the concordance index from 0.58 to 0.75 ((p<0.001)) and improved the area under the receiver operating characteristic curve for daratumumab response from 0.55 to 0.81 ((p<0.001)).

**Discussion:**

ImmunoCast-MM reframes two standard diagnostic examinations as non-invasive profilers of the BMME. The framework may support risk assessment and immunotherapy stratification, particularly in centers without access to high-dimensional flow cytometry.

## 1 Introduction

### 1.1 The bone marrow immune microenvironment as the engine of multiple myeloma progression

Plasma cell clones in multiple myeloma (MM) proliferate only where the bone marrow niche sustains them. Because the bone marrow is both the primary tumor site and the host immune organ, its microenvironment in MM is simultaneously the tumor microenvironment (TME) and the immune microenvironment—a dual role that distinguishes hematologic malignancies from solid tumors, where the TME and the systemic immune compartment are anatomically distinct. Stromal cells, macrophages, CD4 and CD8 T cells, natural killer (NK) cells, regulatory T cells, and myeloid-derived suppressor cells (MDSCs) supply IL-6, IGF-1, APRIL, BAFF, and CXCL12, and the relative composition of those populations governs both disease course and response to therapy (1, 2). From monoclonal gammopathy of undetermined significance to smoldering multiple myeloma and finally to symptomatic disease, the immune compartment of the niche undergoes progressive rewiring. CD8 effector cells acquire exhaustion markers, including PD-1, TIGIT, and LAG-3. NK cells lose NKG2D-dependent cytotoxicity after stress-ligand shedding. Monocytic and granulocytic MDSC populations expand, regulatory T cells accumulate, and bone marrow macrophages polarize to an M2 phenotype (3–5). In older patients, inflammaging superimposes age-dependent lymphoid contraction on these immune lesions, and chronic IL-6 and IL-10 signaling further erodes effector function (5).

Single-cell and mass cytometry studies over the past 5 years have mapped these changes at fine resolution, although at small cohort sizes and in research laboratories (6–8). Each of those studies established what the bone marrow immune microenvironment (BMME) contains. What they leave unanswered is whether the same information can be reached by non-invasive means for cohorts that are too large or too decentralized to receive research-grade immunophenotyping.

### 1.2 The immunotherapy era raises the value of bone marrow microenvironment readouts

The treatment of multiple myeloma has entered an immunotherapy-dominated phase. CD38-directed monoclonal antibodies, bispecific T-cell engagers, and CAR-T cell therapies each depend on an intact patient immune compartment for efficacy, and each exposes a different facet of that compartment to selection pressure. Daratumumab and isatuximab clear tumor plasma cells through antibody-dependent cellular cytotoxicity and trogocytosis, both of which require functional NK populations; patients with pre-treatment NK depletion respond poorly and relapse sooner (9, 10). Bispecific antibodies directed against B-cell maturation antigen (BCMA) (teclistamab and elranatamab) and against GPRC5D (talquetamab) redirect endogenous T cells toward plasma cells. Trial-level analyses of MajesTEC-1, MagnetisMM-3, and MonumenTAL-1 reveal that non-responders cluster along baseline T-cell exhaustion gradients and reduce effector diversity (11–13). Idecabtagene vicleucel and ciltacabtagene autoleucel, in turn, depend on the fitness of autologous T cells at the time of apheresis (14, 15). Immune effector cell adverse events—cytokine release syndrome, immune effector cell-associated neurotoxicity, and prolonged cytopenia—track with baseline myeloid and lymphoid composition across all three therapeutic classes.

None of these signals enter the standard workup. The International Myeloma Working Group (IMWG) response criteria assess tumor burden but not immune competence, and pre-treatment stratification along the effector–suppressor axis remains gated by flow cytometry access. A non-invasive surrogate can change how patients are sorted into therapeutic classes, particularly outside trial centers where high-dimensional immunophenotyping is not routinely available.

### 1.3 Latent immune information in routine diagnostics

Every newly diagnosed multiple myeloma patient undergoes two examinations: a bone marrow aspirate smear and a whole-body ^18^F-FDG PET/CT. Both provide information that the current reading workflow discards.

Cytologists evaluate bone marrow smears to calculate the plasma cell percentage alongside a coarse differential that maps the separation into the myeloid, erythroid, lymphoid, and megakaryocytic lineages. The Wright–Giemsa staining distinguishes lymphoid from myeloid populations with accuracy that matches that of manual differentials. In the same smear slide, granulocyte maturation stages from myeloblast to segmented neutrophil are distinguished, dysplastic morphology and atypical plasma cells are flagged, and spatial clustering among hematopoietic islands is preserved. However, none of these finer-grained signals make it into the final clinical report.

Whole-body ^18^F-FDG PET/CT scans are evaluated using the Italian Myeloma Criteria for PET Use (IMPeTUs) criteria—focal lesion count, maximum standardized uptake value (SUVmax) per lesion, diffuse marrow pattern, and extramedullary disease (16, 17). The same scan also captures spleen uptake as a proxy for systemic immune activation, lymph node distribution, bowel and hepatic uptake, and textural heterogeneity across marrow-containing bones (18). These compartments are ignored in the MM reading workflow, yet they are routinely used in lymphoma and sarcoidosis imaging and encode features of direct immunological interest.

We therefore ask a direct question: is the immunological information latent in these two tests sufficient, once decoded by modern deep learning, to reconstruct a clinically useful view of the bone marrow immune microenvironment?

### 1.4 Study objectives and contributions

**Figure 1:**
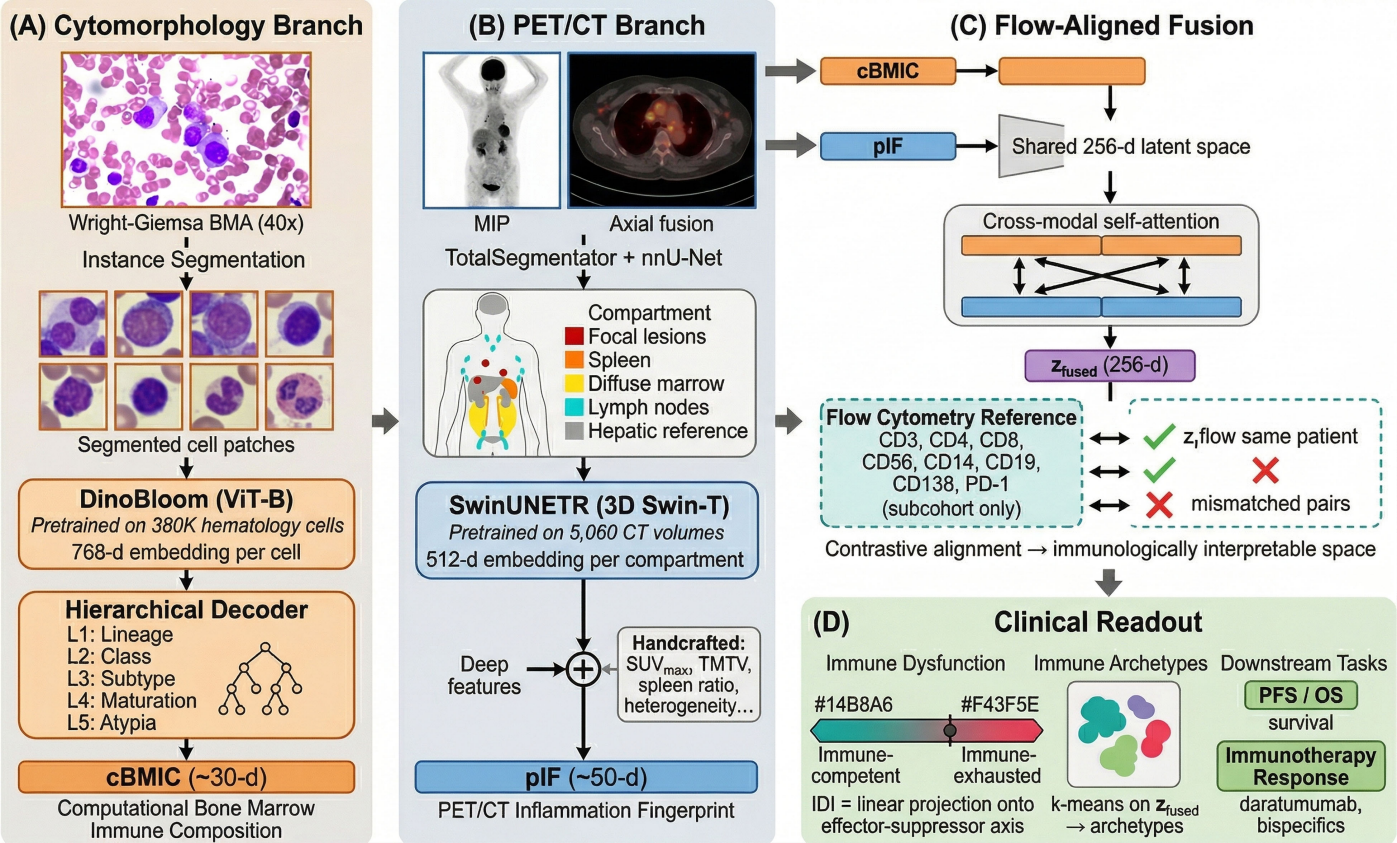
ImmunoCast-MM framework for non-invasive bone marrow immune microenvironment decoding in multiple myeloma. **(A)** Cytomorphology branch. Wright–Giemsa-stained bone marrow aspirate smears enter an instance segmentation network that isolates individual cells. Each cell crop passes through DinoBloom, a hematology-specific vision transformer pretrained on 380,000 single-cell images (19), which yields a 768-dimensional embedding. A hierarchical decoder with condition-dependent heads classifies each embedding across five levels: lineage (lymphoid, myeloid, erythroid, plasma, and other lineages), class, subtype, maturation stage, and atypia flag. Patient-level aggregation produces the Computational Bone Marrow Immune Composition (cBMIC) vector. **(B)** PET/CT branch. Each ^18^F-FDG PET/CT scan undergoes SUV normalization, compartment segmentation using TotalSegmentator (20), a fine-tuned nnU-Net, and compartment-level feature extraction that involves the tumor, spleen, lymph nodes, diffuse marrow, and a hepatic reference. A 3D Swin Transformer pretrained on 5,050 CT volumes (21) supplies a 512-dimensional embedding per compartment. Concatenation with handcrafted metrics yields the PET/CT inflammation fingerprint (pIF) vector. **(C)** Cross-modal fusion. cBMIC and pIF are projected into a shared 256-dimensional latent space, and self-attention between the two modality tokens produces a fused patient embedding. A contrastive objective aligns this embedding with a flow cytometry reference panel on a subcohort, which converts the joint space into an immunologically interpretable coordinate system. **(D)** Clinical readout. A linear projection of the fused embedding onto a learned effector–suppressor axis defines the Immune Dysfunction Index (IDI), unsupervised clustering discovers immune archetypes, and task-specific heads support downstream analyses of progression-free survival and immunotherapy response.

In this work, we propose ImmunoCast-MM, a cross-modal deep learning framework that addresses four related aims (**Figure 1**).

First, a hierarchical cell decoder classifies every cell on a Wright–Giemsa-stained bone marrow aspirate smear into a five-level immune taxonomy—lineage, class, subtype, maturation stage, and atypia—and aggregates the results into a Computational Bone Marrow Immune Composition (cBMIC) vector whose resolution exceeds that of manual differentials.

Second, the framework extends ^18^F-FDG PET/CT analysis beyond tumor regions by deriving a multi-organ inflammation fingerprint that involves spleen, lymph node, diffuse marrow, and hepatic reference compartments and combines handcrafted metabolic metrics with a 3D foundation model embedding.

Third, a contrastive fusion module aligns the two modality outputs in a shared embedding space anchored to a flow cytometry reference panel on a subcohort, which yields a biologically interpretable Immune Dysfunction Index along an effector–suppressor axis.

Fourth, we evaluate whether the decoded signals correlate with flow cytometric immune subsets, associate with progression-free survival, stratify response to daratumumab-containing induction, and describe a hypothesis-generating later-line bispecific antibody subset without treating it as a discrimination analysis.

We are not aware of prior work that attempted to use a computational taxonomy decoder for the bone marrow immune microenvironment from routine cytomorphology, an ^18^F-FDG PET/CT inflammation fingerprint designed for immune rather than tumor readouts in multiple myeloma, or a flow cytometry-aligned cross-modal latent space for the disease. The clinical demonstration targets immunotherapy response stratification, an issue that prior imaging or pathology artificial intelligence systems for MM have not addressed.

## 2 Related work

### 2.1 Immunophenotyping of the multiple myeloma bone marrow microenvironment

High-dimensional approaches to the MM immune niche have matured over the past decade. EuroFlow standardization delivered reproducible plasma cell identification and minimal residual disease measurement across European centers (22). Mass cytometry panels that cover effector, exhausted, and suppressor subsets within the same tube revealed the co-emergence of exhausted CD8 populations and monocytic suppressor cells during progression (1, 6). Single-cell RNA sequencing atlases traced the evolution of the immune compartment from monoclonal gammopathy of undetermined significance to symptomatic disease, exposed precursor states of exhaustion years before relapse, and associated regulatory T-cell expansion with cytogenetic risk (3–5). Spatial transcriptomic studies have added anatomic context and shown that proximity between plasma cells and exhausted CD8 neighborhoods predicts progression more reliably than bulk composition alone (7, 8). Together, these methods define what the BMME contains. Their translation into routine workflows remains bounded by cost, fresh-sample requirements, and the absence of standardized analysis pipelines.

### 2.2 Computational pathology for hematologic malignancies

Computational cell recognition has moved from handcrafted shape descriptors to deep convolutional neural networks to hematology-specific foundation models. Matek and colleagues trained cell classifiers for acute myeloid leukemia on peripheral blood and bone marrow smears that approach expert agreement across 20 cell classes (23, 24). DinoBloom extended this line through self-supervised pretraining on 380,000 hematology cell images and produced embeddings that outperform ImageNet-pretrained backbones across several downstream tasks (19). Recent work has applied these embeddings to morphology-to-molecular prediction in acute leukemia and multiple myeloma (25–27). No prior work has repurposed these tools for immune microenvironment decoding; every existing system is tuned for tumor or dysplasia detection rather than for immune composition.

### 2.3 Deep learning immune profiling in solid tumor histology

Tumor-infiltrating lymphocyte (TIL) scoring from hematoxylin and eosin (H&E) whole-slide images now serves as a scalable readout of the tumor immune microenvironment in solid tumors (28–30). Deep learning models predict immune gene expression and immune subtypes from morphology alone across multiple cancer types (31). Architectures such as HoVer-Net and CERBERUS resolve cell types and spatial immune architecture in routine H&E (32). None of these systems transfer directly to Wright–Giemsa-stained bone marrow aspirate smears: the cell taxonomy differs, the staining protocol is different, and in marrow, the entire field contains immune and hematopoietic cells rather than a small infiltrate within an epithelial background.

### 2.4 ^18^F-FDG PET/CT readouts beyond tumor burden

Standard PET/CT metrics in MM focus on tumor: total metabolic tumor volume (TMTV), total lesion glycolysis (TLG), SUVmax per lesion, and focal lesion count (33–35). Those metrics drive prognostic models and response assessment, and they underpin the IMPeTUs reporting framework (16). In lymphoma and inflammatory disease research, non-tumor compartments have received separate attention: spleen SUV as a proxy for systemic immune activation, diffuse marrow uptake as a reactive hematopoiesis signal, and lymph node uptake as a reflection of immune engagement (18, 36). PET radiomics adds texture and heterogeneity descriptors that capture spatial variation within each compartment (37, 38). These inflammation-aware readouts have not been applied to multiple myeloma, and no prior study has fused them with cytomorphology for immune inference.

### 2.5 Multi-modal medical AI fusion

Cross-modal architectures in oncology have shifted from simple concatenation to co-attention to contrastive alignment. Pathomic Fusion and Multimodal Co-Attention Transformer (MCAT) demonstrated that histology–genomics co-attention outperforms late fusion for survival prediction across cancer types (39, 40). Contrastive learning, established for general vision by Contrastive Language–Image Pre-training (CLIP) (41), now underpins medical foundation models that align images with clinical text or with molecular profiles (42, 43). No prior fusion work has aligned the joint embedding space with an immune reference measurement. We adopt this strategy as the mechanism through which cross-modal decoding becomes immunologically interpretable.

## 3 Materials and methods

### 3.1 Study cohort, ethics, and sample definitions

**Figure 2:**
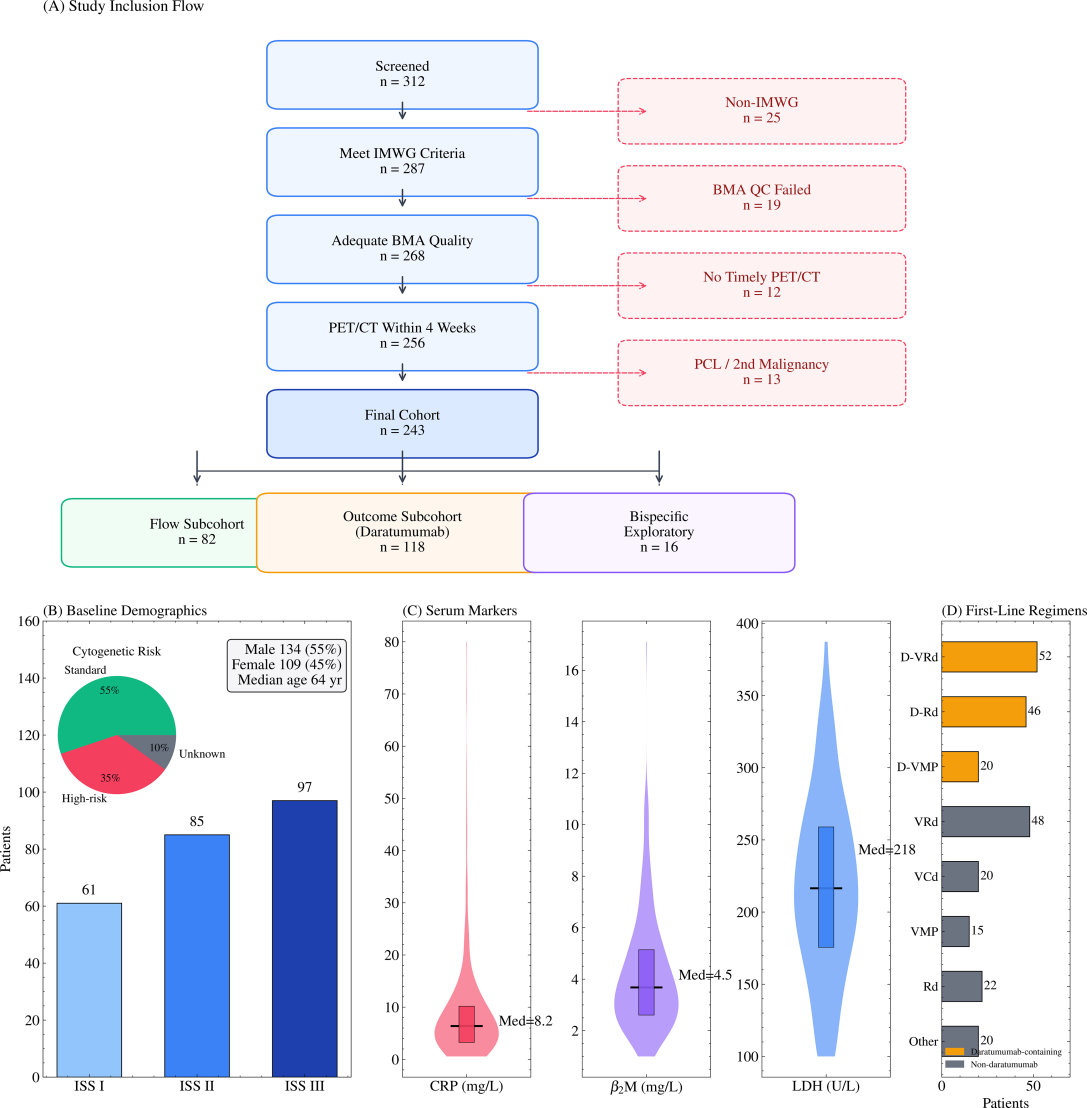
Cohort structure, baseline demographics, and immune starting point. **(A)** Consolidated Standards of Reporting Trials (CONSORT)-style inclusion flow diagram that traces the full retrospective cohort through quality control and then identifies the flow cytometry subcohort used for cross-modal alignment and the outcome subcohort used for immunotherapy response analysis. **(B)** Distribution of baseline demographics, including age, sex, International Staging System (ISS) stage, and cytogenetic risk category. **(C)** Distribution of baseline serum inflammatory markers—C-reactive protein, β_2_-microglobulin, and lactate dehydrogenase—as a first view of the immune starting point prior to deep learning-based decoding. **(D)** First-line treatment regimens ordered by frequency, with the daratumumab-containing subcohort highlighted for later use in response analysis.

A retrospective cohort of newly diagnosed multiple myeloma patients diagnosed between January 2018 and December 2023 at the First Affiliated Hospital of Ningbo University was assembled. Eligible patients met IMWG diagnostic criteria (44), had at least one baseline bone marrow aspirate smear with four or more ×40 fields of adequate staining and focus, underwent baseline ^18^F-FDG PET/CT within 4 weeks of aspiration, and had documented first-line therapy with available follow-up. Patients with plasma cell leukemia, concurrent second malignancies, or smears that failed quality control were excluded. Cohort demographics, staging, cytogenetic risk category, and treatment regimens are summarized in **Figure 2**.

Two nested subcohorts were used for the immunological analyses. The flow cytometry subcohort consisted of patients with a baseline multi-parameter flow cytometry reference panel that covered CD3, CD4, CD8, CD14, CD19, CD25, CD38, CD45, CD56, CD127, CD138, and HLA-DR at minimum. These samples anchored the cross-modal latent space to an immune reference. The outcome subcohort consisted of patients who received daratumumab-containing induction regimens—including D-VRd, DRd, and D-VMP—and served as the target for immunotherapy response analysis. Of the 82 patients with flow cytometry reference panels, 58 received daratumumab-containing induction and were therefore included in the outcome subcohort consisting of 118 patients, while the remaining 24 patients received non-daratumumab regimens; conversely, 60 of 118 outcome subcohort patients had no flow cytometry data and never contributed to the contrastive loss or to the Immune Dysfunction Index (IDI) axis fit. A smaller exploratory subset of 16 patients who received later-line bispecific antibody therapy (BCMA × CD3, *n* = 9; GPRC5D × CD3, *n* = 7) was included for hypothesis-generating analysis only.

**Figure 3:**
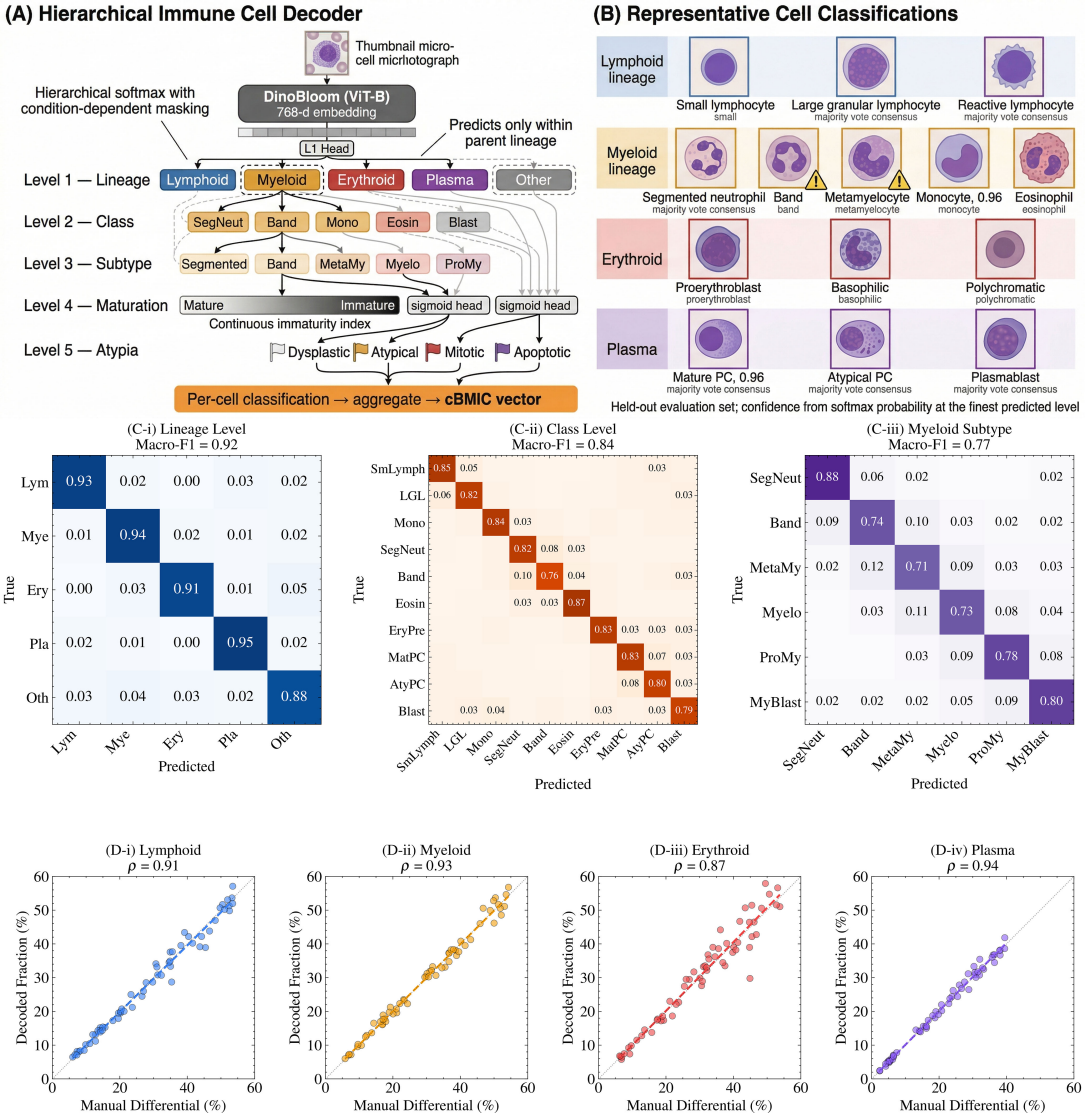
Hierarchical immune cell decoder performance. **(A)** Decoder architecture diagram, with five semantic levels and condition-dependent classification heads that operate on DinoBloom embeddings. **(B)** Example cell patches for each fine-grained class, annotated with model confidence and with the majority label from the three consensus hematologists. **(C)** Confusion matrices at the lineage, class, and subtype levels on the held-out evaluation set. **(D)** Agreement between decoded lineage fractions and manual differential counts from the hospital laboratory across the evaluation patients.

The study received ethical approval from the Institutional Review Board of the First Affiliated Hospital of Ningbo University, with a waiver of informed consent for retrospective use of de-identified imaging and smear data. Written informed consent for the publication of de-identified clinical images was obtained from the patients whose imaging and smear data appear in **Figures 1**, **3**. All procedures conformed to the principles of the Declaration of Helsinki and to institutional data protection requirements.

### 3.2 Bone marrow aspirate cytomorphology pipeline

Wright–Giemsa-stained bone marrow aspirate smears were digitized on a Jiangfeng Bio KF PRO 120 whole-slide scanner at ×40 magnification (0.25 μm/pixel; sRGB color profile). Each field passed through five stages: stain normalization, cell segmentation, foundation feature extraction, hierarchical classification, and patient-level aggregation.

Stain normalization used the Macenko method, which reduces chromatic variation introduced by different staining batches and scanner hardware. Cell segmentation used an instance segmentation model based on Cellpose-SAM, fine-tuned on annotated bone marrow crops, to produce per-cell masks and 128 × 128 patches centered at each segmented nucleus. Segmentation quality was evaluated on a 250-cell held-out set among 10 patients who were excluded from the fine-tuning process: detection precision of 0.94, recall of 0.91, F1 of 0.92 at an intersection-over-union (IoU) threshold of 0.5, with median per-cell IoU of 0.87 (interquartile range 0.81–0.91), a split-cell rate of 2.8%, and a merged-cell rate of 1.6% (**Appendix Table 4**). To test the propagation of segmentation error to downstream estimates, patient-level aggregation was re-run under ±2 pixel cell-mask dilation and erosion; per-class cBMIC frequencies shifted by less than 0.5 percentage points, and the IDI shifted by no more than 0.02 from baseline. Each patch was entered into the DinoBloom-B model, a self-supervised vision transformer pretrained on 380,000 hematology cell images, which produced a 768-dimensional embedding per cell (19).

Embeddings were then passed into the hierarchical immune cell decoder, which organizes classification across five semantic levels. Level 1 assigns each cell to a lineage: lymphoid, myeloid, erythroid, plasma, or other lineages. Level 2 refines the lineage into a class such as small lymphocyte, large granular lymphocyte, monocyte, granulocyte, erythroid precursor, plasma cell, megakaryocyte, or blast. Level 3 identifies subtypes within each class: segmented neutrophil, band, metamyelocyte, myelocyte, promyelocyte, or myeloblast within the myeloid line; or mature, reactive, or plasmablastic morphology within the plasma cell line. Level 4 encodes a maturation position that becomes a continuous immaturity index per lineage. Level 5 flags atypia, including dysplastic morphology, atypical plasma cells, mitotic figures, and apoptotic forms.

The decoder uses a hierarchical softmax with condition-dependent masking so that each level predicts only within the class chosen at the previous level. Focal loss with class re-weighting counters the heavy imbalance in marrow cell frequencies, and label smoothing limits overconfidence on ambiguous cells (24).

Patient-level aggregation takes the frequency histogram of predicted classes across all cells in all fields per patient and yields a cBMIC vector of approximately 30 dimensions. Five derived indices summarize this vector for downstream analysis: the lymphoid-to-myeloid ratio, the plasma cell burden, the atypical plasma cell fraction, the myeloid immaturity index (a proxy for myeloid-derived suppressor-like expansion), and the lymphoid reactivity index (a proxy for adaptive immune activation).

Training supervision drew on three sources. Public single-cell annotations from the Matek marrow dataset provided the initial label set (24). Three hematologists at our center independently annotated a held-out sample of 5,124 cells stratified across lineages, and the majority vote resolved disagreements. Pairwise inter-rater agreement reached Fleiss’ *κ* = 0.81 at the lineage level and *κ* = 0.72 at the class level; the lowest agreement occurred between metamyelocyte and band classes (*κ* = 0.58), which mirrors the dominant confusion mode of the decoder. Self-training on non-study scans supplied pseudo-labels that constituted approximately 18% of the final training set. Only cells whose maximum softmax probability exceeded 0.95 after two rounds of teacher–student refinement were retained as pseudo-labeled examples; an ablation without pseudo-labels reduced the class-level macro-F1 score by 0.03 (from 0.84 to 0.81), which confirmed a modest but consistent benefit. Additionally, because this threshold is a filtering rule rather than a proof of calibration, the pseudo-labeled cells were characterized directly. Their per-class frequency distribution did not differ detectably from that of expert-annotated cells (*χ*^2^ test, *p* = 0.41), and the DinoBloom-embedding centroid distance between the pseudo and expert cells of the same class was small (Cohen’s *d* ≈ 0.18 and across 0.84 classes). A confidence threshold sweep over {0.90, 0.95, 0.99} yielded macro-F1 scores of 0.83, 0.84, and 0.84, respectively, placing the chosen 0.95 threshold on a plateau rather than at a peak.

### 3.3 PET/CT multi-organ inflammation fingerprint pipeline

All scans were acquired on a Siemens Biograph mCT PET/CT scanner. Patients fasted for at least 6 hours and had blood glucose below 150 mg/dL, received a weight-based dose of 5.55 MBq/kg of ^18^F-FDG, and were imaged 60 minutes after injection. Reconstruction used time-of-flight and point-spread function modeling with ordered-subsets expectation maximization (OSEM) (4 iterations × 21 subsets) and a 5-mm body Gaussian post-filter; CT reconstruction was performed with a 3-mm slice thickness for body regions and 1.5 mm for pulmonary regions. Each PET/CT scan underwent SUV normalization to body weight, spatial resampling to 2 × 2 × 2 mm isotropic voxels, intensity windowing for the CT channel, and rigid registration between PET and CT volumes. TotalSegmentator delineated anatomical compartments, which included the spleen, liver, kidneys, cervical and abdominal lymph node regions, and bowel (20). A fine-tuned nnU-Net segmented bone marrow regions across the axial skeleton, pelvis, and proximal long bones (45), and clinician-reviewed contours defined focal lesions on the tumor subset.

Each compartment yielded a feature set that combined handcrafted metrics with a foundation model embedding. For the tumor compartment, the handcrafted features included TMTV, TLG, SUVmax, SUVpeak, lesion count, lesion volume distribution, and a spatial spread index that captures the physical extent of disease across the skeleton. For the spleen compartment, SUVmean, SUVmax, spleen-to-liver SUV ratio, and volumetric distribution were recorded. The lymph node compartment contributed the number of PET-avid nodes above SUV 2.5, the highest nodal SUVmax, and the distribution of uptake across the cervical, axillary, mediastinal, and abdominal regions. The diffuse marrow compartment supplied marrow-to-liver SUV ratio, marrow SUV heterogeneity through gray-level co-occurrence matrix entropy and uniformity, and a reactive-versus-infiltrative pattern score defined as the ratio of voxels whose SUV exceeds the liver reference by more than one standard deviation to all marrow voxels, where high values indicate diffuse infiltration and low values suggest reactive hematopoiesis. The hepatic reference compartment confirmed the stability of normalization across slices, and whole-body descriptors captured peritumoral-versus-distal SUV, bowel uptake relative to fasting state, and muscle background.

Each compartment volume also passed through SwinUNETR, a 3D Swin Transformer pretrained on 5,050 CT volumes (21), which produces a 512-dimensional embedding. The embedding and the handcrafted features were concatenated into a PET/CT inflammation fingerprint (pIF) vector of approximately 50 dimensions per patient.

### 3.4 Cross-modal fusion and the immune dysfunction index

The fusion block takes the cBMIC vector and the pIF vector as input, projects each through an encoder head into a shared 256-dimensional latent space, and applies self-attention across the two modality tokens to produce a fused patient embedding. The architecture follows established cross-modal transformer designs (39, 40), with one change whose role is central: the training objective is contrastive alignment with a flow cytometry reference rather than direct outcome supervision.

Within the flow cytometry subcohort, each patient received a reference immune vector computed from panel fractions—CD4 helper, CD8 effector, CD56 NK, CD14 monocytic, CD19 B, CD138 plasma, and regulatory T-cell fractions, where panel composition allowed. Because clinical panels evolved over the enrollment period, 14 of 82 patients with flow cytometry reference panels had abbreviated panels, which omitted one or more T-cell subset markers or lacked full NK phenotyping resolution (9 in the 49-patient training partition and 5 in the 33-patient held-out subset, against 40 and 28 full-panel patients, respectively); for these patients, the reference vector was truncated to the available dimensions, and the contrastive loss was computed only over matched dimensions with per-dimension weighting to preserve gradient balance. No imputation was applied. A CLIP-style contrastive loss pulled the fused embedding of each patient toward their own reference vector and pushed it away from mismatched references (41). Although the contrastive head was trained on the 49 patients of $T_{49}$, the reference vector was dense across roughly 12 flow-panel dimensions per patient, so the alignment objective was on the order of 49 × 12 ≈ 600 per-dimension scalar constraints rather than 49 binary positive–negative pairs. These flow dimensions are correlated and do not increase the number of independent patients, but they provide continuous multi-dimensional supervision rather than a single class label per patient. This constraint trains the fused space to encode immune composition directly rather than whatever signal happens to predict a downstream outcome.

After alignment, the IDI was defined as a fixed linear projection of the fused embedding onto a flow-derived effector–suppressor axis. The axis was fitted using ridge regression on the 49 training partition patients with flow cytometry reference panels only (denoted as $T_{49}$). For each such patient *i*, we compute a continuous composite immune score

(1) $$s_{i}=z\left(\text{CD}8_{\text{eff}}\%\right)+z\left(\text{CD}56\;\text{NK}\%\right)-z\left(\text{CD}14\;\text{mono}\%\right)-z\left({\text{T}}_{\text{reg}}\%\right),$$

where z(·) denotes per-dimension z-scoring across $T_{49}$, and CD8 effector cells are operationalized from the CD3^+^CD8^+^ gate, NK cells from the CD3^−^CD56^+^ gate, monocytes from the CD14^+^ gate, and regulatory T cells from the CD4^+^CD25^+^CD127^low^ gate, where available. Dimensions missing from abbreviated panels are dropped from the sum, so no imputation is required. The axis is then obtained as

(2) $$w^{*},b^{*}=\text{arg}\underset{w,b}{\min}\sum_{i\in T_{49}}{\left(w^{⊤}f_{\theta }\left(x_{i}\right)+b-s_{i}\right)}^{2}+\text{λ}{\left||w|\right|}_{2}^{2},$$

where $f_{\theta }\left(\cdot \right)$ is the fused encoder and the regularizer λ is selected using an inner fivefold cross-validation on $T_{49}$. The released axis $\hat{w}=w^{*}/{\vdots w^{*}\vdots }_{2}$ is a single unit vector in the 256-dimensional latent space, and the intercept is rescaled as $\hat{b}=b^{*}/{\left||w^{*}|\right|}_{2}$ to keep the projection on the same scale. The axis and intercept are fixed once at the end of training and are not adapted per patient. The IDI for any patient *x* is then $\text{IDI}\left(x\right)={\hat{w}}^{⊤}f_{\theta }\left(x\right)+\hat{b}$. Only the 49 patients in $T_{49}$ were used to construct the composite immune score and fit the effector–suppressor axis in Equations 1 and 2. The 33 held-out patients with flow cytometry data were reserved exclusively for validation, whereas the 161 patients without flow cytometry data were used only for downstream IDI-based analyses. By construction, one end of the axis corresponds to immune-competent profiles—elevated CD8 effector and NK content alongside low monocytic and regulatory T-cell content—and the other end to immune-exhausted profiles. The IDI is reported as a continuous score and, for stratified analyses, as tertiles.

For archetype discovery, we apply unsupervised clustering (*k*-means with *k* ∈ {2,3,4}) to the fused embeddings across the full cohort, select *k* by silhouette score, and characterize each cluster by its dominant cytomorphologic and metabolic features.

### 3.5 Training protocol

The framework was implemented in PyTorch, with MONAI for 3D PET/CT processing and timm for vision transformer backbones. Optimization used AdamW with a cosine schedule and warm restarts, base learning rate of 1 × 10^−4^, weight decay of 1 × 10^−2^, and gradient clipping at norm of 1.0. Bone marrow crops received stain augmentation in HED space, color jitter, random rotation, and elastic deformation. PET/CT volumes received affine augmentation, SUV jitter of ±5%, and random compartment dropout. Regularization included a dropout of 0.2 in the fusion encoder and early stopping on the flow–alignment correlation measured on a validation split.

**Figure 4:**
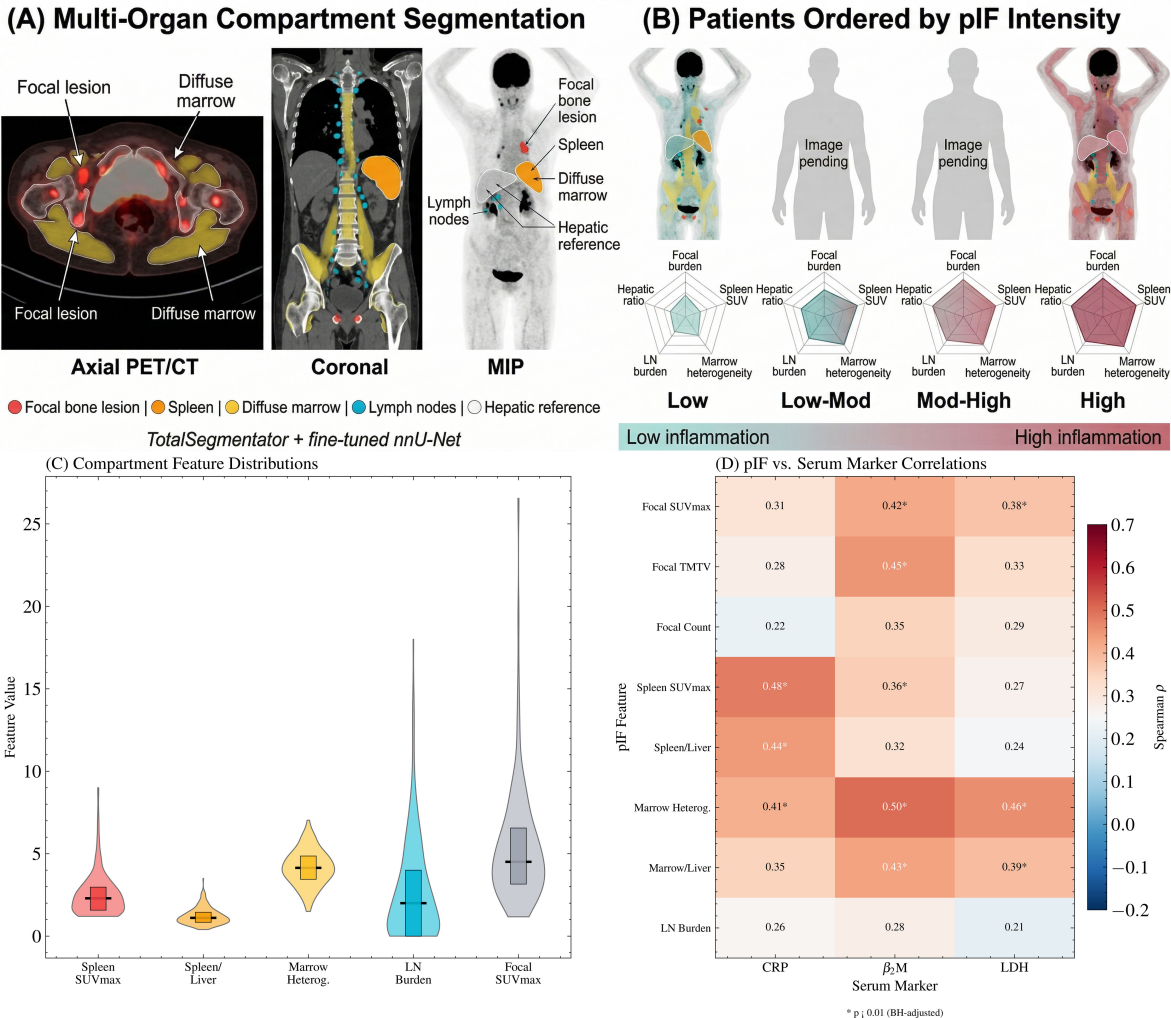
PET/CT multi-organ inflammation fingerprint. **(A)** Representative compartment segmentation on axial, coronal, and maximum-intensity-projection views, with tumor, spleen, lymph node, and diffuse marrow regions highlighted. **(B)** Four representative patients ordered by increasing fingerprint intensity, with corresponding compartment overlays and radar-chart summaries. **(C)** Per-patient distributions of compartment features, including spleen SUVmax, spleen-to-liver ratio, diffuse marrow heterogeneity, and lymph node burden. **(D)** Spearman’s rank correlations between the inflammation fingerprint dimensions and serum inflammatory markers (C-reactive protein, β_2_-microglobulin, and lactate dehydrogenase).

Cross-validation followed a patient-stratified fivefold scheme with an external hold-out subset. Within the flow cytometry subcohort (*n* = 82), 49 patients anchored the contrastive alignment loss during training, and 33 were withheld as a dedicated validation set that never contributed to the contrastive objective in any fold. This partition was fixed across all five folds to prevent information leakage: alignment correlations reported in **Figure 4** are computed exclusively on the 33-patient held-out subset. For the remaining 161 patients without flow cytometry data, contrastive terms were not applicable; these patients contributed only through the classification and outcome objectives. The IDI axis (Equation 2) was fitted on the same 49-patient training partition once contrastive training had converged and was held fixed across all evaluation folds; no fold or patient outside $T_{49}$ contributed to the axis fit. Training was run on a single NVIDIA A100 GPU for approximately 72 hours end-to-end.

### 3.6 Statistical analysis and evaluation

The evaluation spanned three layers. The classification quality of the cytomorphology decoder was reported as per-class F1, macro-F1, and top-1 accuracy at each hierarchical level, with confusion matrices for the most confusable classes. Agreement with manual differentials from the hospital laboratory was expressed as Spearman’s rank correlation across major lineages.

Alignment quality with flow cytometry was measured using Spearman’s rank correlation between decoded fractions and their flow cytometric counterparts on the validation subset of the flow cytometry subcohort, with bootstrap 95% confidence intervals and the Benjamini–Hochberg adjustment across the panel dimensions. The pIF-to-serum inflammation relationship was investigated using Spearman’s rank correlations against C-reactive protein, β_2_-microglobulin, and lactate dehydrogenase.

Clinical association was assessed using the Cox proportional hazards regression for progression-free survival, with the Kaplan–Meier curves stratified by IDI tertile and log-rank tests. Overall survival (OS) was pre-specified as a secondary endpoint; however, data maturity at the current follow-up was insufficient for reliable estimation, and OS results were therefore deferred to a future update. Response analysis was performed using logistic regression of objective response to daratumumab-containing induction against the IDI, adjusted for International Staging System (ISS) stage, cytogenetic risk category, and age. Additionally, because the assignment of daratumumab in this retrospective cohort was not random, a propensity score model was fitted for entry into the daratumumab-containing outcome subcohort using age, ISS stage, cytogenetic risk, diagnosis era, Eastern Cooperative Oncology Group (ECOG) performance status, and renal function; the IDI–outcome Cox and logistic models were re-fit under inverse probability of selection weighting (IPSW). The E-value of the IDI hazard ratio was also reported to quantify the strength of unmeasured IDI–outcome confounding required to nullify the association. Additionally, because frontline MM therapy at our institution evolved substantially over the 2018–2023 enrollment window, the Cox model was adjusted for diagnosis era as a direct covariate (binary, 2018–2020 vs. 2021–2023), and a full-cohort regimen-stratified sensitivity Cox model was reported across the major induction regimens (VRd, D-VRd, DRd, D-VMP, and other regimens) to assess residual era effects beyond the broad daratumumab versus non-daratumumab contrast. Model discrimination was measured using the concordance index for Cox models and the AUC for logistic response models. C-index confidence intervals were computed using 2,000 bootstrap resamples, improvement in nested Cox models was assessed using the likelihood ratio test, and AUC comparisons were performed using the DeLong test (46), with bootstrap confidence intervals, where reported. All analyses were conducted in Python (lifelines, scikit-learn, and scipy) and R (survival, survminer, pROC, and ggplot2).

### 3.7 Explainability and biological interpretation

The framework exposes three forms of explanation. Per-patient summary reports list the top contributing cell classes and metabolic compartments, the placement of the patient along the effector–suppressor axis, and the assigned archetype. Cross-modal co-attention maps show which PET/CT compartment features activate strongly when specific cell composition signals are present, and vice versa. Biological sanity checks compare decoded signals against known immunology: patients with high myeloid immaturity are expected to show elevated spleen SUVmax and diffuse marrow uptake, and lymphoid-depleted patients are expected to cluster with exhausted-phenotype profiles in the flow cytometry reference. These checks serve both as a validation and as a mechanism to reveal unexpected failure modes.

## 4 Results

### 4.1 Cohort characteristics and immune baseline

**Figure 2** summarizes the cohort characteristics. Patients spanned the range of newly diagnosed MM presentations, with representation across ISS stages and the standard cytogenetic risk categories. The flow cytometry subcohort covered the same range at a reduced sample size, and the outcome subcohort was dominated by daratumumab-containing induction regimens consistent with contemporary first-line practice at our institution. Baseline serum inflammatory markers spanned the expected range of newly diagnosed disease and provided a starting point against which the decoded PET/CT inflammation fingerprint can be compared. The smaller bispecific antibody subset covered only later-line patients and was treated as exploratory throughout the analyses that followed.

### 4.2 Cytomorphology decoder performance

**Figure 5:**
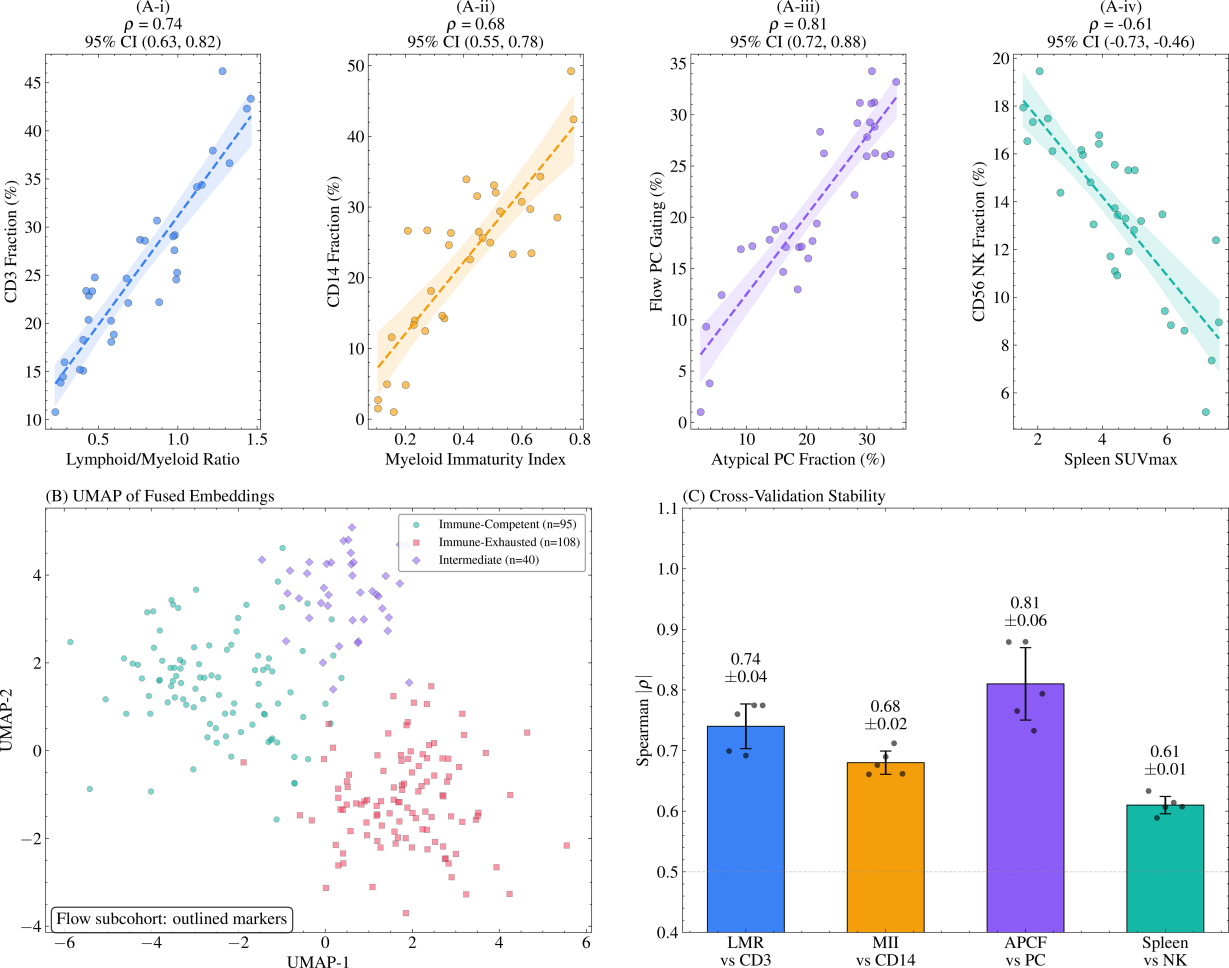
Flow cytometry-aligned validation of decoded cytomorphologic and raw imaging signals. **(A)** Pairwise scatterplots between decoded cytomorphologic indices or raw spleen SUVmax and matched flow cytometric fractions on the validation subset of the flow cytometry subcohort: lymphoid-to-myeloid ratio versus CD3, myeloid immaturity index versus CD14, atypical plasma cell fraction versus flow cytometry gating for plasma cells, and spleen SUVmax versus CD56 NK fraction. Correlation coefficients and bootstrap 95% confidence intervals are reported. **(B)** UMAP projection of fused cross-modal embeddings colored by flow-derived archetype labels, which illustrates the learned alignment geometry. **(C)** Stability of flow–alignment correlations across the five cross-validation folds.

The hierarchical decoder yielded lineage-level assignments that agreed with manual differentials read by hospital cytologists on the held-out evaluation set. Fine-grained class-level assignments performed the best for plasma cells, small lymphocytes, and segmented neutrophils—the classes with the most distinctive morphology. Subtype-level assignments within the myeloid line retained interpretable accuracy across maturation stages. Confusion between adjacent stages, metamyelocyte versus band in particular, remained the dominant error mode. Atypia flagging identified dyspoietic and atypical plasma cell morphologies at rates consistent with the reference annotations of the three consensus readers. **Figure 5** reports confusion matrices at each hierarchical level, example patches with model confidence per class, and agreement between decoded lineage fractions and manual differentials. **Table 1** provides the corresponding per-class precision, recall, and F1 scores at each hierarchical level.

**Table 1:** Per-class classification metrics of the hierarchical immune cell decoder on the held-out evaluation set.

Level	Class	Precision	Recall	F1	Support
Lineage	Lymphoid	0.94	0.93	0.93	2,862
	Myeloid	0.90	0.94	0.92	7,884
	Erythroid	0.95	0.91	0.93	1,428
	Plasma cell	0.93	0.95	0.94	1,512
	Other	0.89	0.88	0.89	714
	Macro avg.	0.92	0.92	0.92	14,400
Class (10-way)	Macro avg.	0.85	0.82	0.84	12,600
Myeloid subtype	Seg. neutrophil	0.86	0.88	0.87	3,024
	Band	0.76	0.74	0.75	1,890
	Metamyelocyte	0.72	0.71	0.71	1,134
	Myelocyte	0.73	0.73	0.73	966
	Promyelocyte	0.79	0.78	0.78	504
	Myeloblast	0.81	0.80	0.80	366
	Macro avg.	0.78	0.77	0.77	7,884

The class-level row reports the macro average across the full 10-way taxonomy; per-class detail for that level is shown in the confusion matrix of **Figure 5C**.

### 4.3 PET/CT inflammation fingerprint characterization

Compartment feature distributions revealed patient-to-patient variability that tumor-centric reading discards. Spleen SUVmax spanned a wide range. A subset of patients showed distinctly elevated uptake consistent with immune activation rather than disease infiltration. The diffuse marrow compartment differentiated patients whose uptake profile suggests reactive hematopoiesis from those with an infiltrative pattern. Lymph node metrics added a third axis of variation, one that is meaningful even among patients without bulky nodal involvement. Spearman’s rank correlations against serum C-reactive protein, β_2_-microglobulin, and lactate dehydrogenase confirmed that the inflammation fingerprint tracked systemic inflammation rather than tumor burden alone. **Figure 3** displays the compartment segmentation examples, feature distributions, serum correlations, and a set of representative patients ordered from low to high fingerprint intensity.

### 4.4 Flow cytometry-aligned validation of decoded and imaging signals

The flow cytometry subcohort provided the alignment anchor. **Figure 4** presents pairwise scatterplots between cytomorphologic indices and their flow cytometric counterparts on the 33-patient held-out validation set. The decoded lymphoid-to-myeloid ratio tracked the flow cytometry-determined CD3 fraction (*ρ* = 0.74; 95% CI 0.63–0.82), the myeloid immaturity index tracked the CD14 monocytic fraction (*ρ* = 0.68; 95% CI 0.55–0.78), and the atypical plasma cell fraction tracked flow cytometry gating for plasma cells (*ρ* = 0.81; 95% CI 0.72–0.88). These three comparisons evaluated decoder-derived cytomorphologic summaries against flow cytometry on unseen patients; they were flow–alignment validation results, not IDI–flow correlations, nor clinical outcome tests. Spleen SUVmax, by contrast, is a raw PET/CT feature rather than a decoded cytomorphology or IDI component; it tracked the CD56 NK fraction in the opposing direction (*ρ* = −0.61; 95% CI −0.73 to −0.46). All four correlations remained significant after the Benjamini–Hochberg correction across the panel dimensions (adjusted *p* < 0.001 for each). Restricting the same comparison to the 28 held-out patients with complete flow panels generated *ρ* = 0.72, 0.66, 0.80, and −0.63 for the same four pairs, all within the bootstrap 95% CIs of the headline values. A Uniform Manifold Approximation and Projection (UMAP) of fused embeddings colored by flow-derived labels illustrates the learned alignment geometry. Held-out alignment quality remained stable across cross-validation folds, which indicates that the contrastive objective recovered a reference-space mapping rather than a fold-specific artifact.

### 4.5 Bone marrow immune archetypes and clinical stratification

Unsupervised clustering of fused embeddings yielded two dominant archetypes—immune-competent and immune-exhausted—with an intermediate cluster at higher values of *k*. The immune-exhausted cluster carried elevated myeloid immaturity, reduced lymphoid fraction, elevated spleen SUVmax, and elevated diffuse marrow heterogeneity; the immune-competent cluster showed the opposing profile.

At a median follow-up of 26 months (interquartile range 14–38), 79 of 118 patients in the outcome subcohort experienced progression or death (event rate 67%). The Kaplan–Meier curves for progression-free survival separated the two archetypes and showed a further gradient across IDI tertiles. Response rates to daratumumab-containing induction decline from the immune-competent to the immune-exhausted archetype. After adjustment for ISS stage, cytogenetic risk, and age in a Cox model with 79 events across six covariates (13.2 events per variable), the IDI effect remained intact. Under inverse probability of selection weighting based on the propensity to enter the daratumumab-containing outcome subcohort, the IDI hazard ratio for progression-free survival (PFS) was 1.95 (95% CI 1.18–3.22), within the confidence interval of the unweighted estimate; the corresponding E-value was 3.3, meaning that an unmeasured confounder would need an association of hazard ratio strength ≥3.3 with both the IDI and PFS, beyond the measured covariates, to fully account for the IDI effect. **Appendix Table 3** reports the daratumumab versus non-daratumumab baseline comparison. Adding diagnosis era (2018–2020 vs. 2021–2023) as a direct Cox covariate gave an IDI hazard ratio for PFS of 2.05 (95% CI 1.30–3.24), comparable to the IPSW estimate; in a full-cohort sensitivity analysis, a regimen-stratified Cox model fitted across VRd, D-VRd, DRd, D-VMP, and other induction regimens gave stratum-specific IDI hazard ratios in the range of 1.7–2.3, all directionally consistent with the pooled estimate. Restricting the analysis to the 60 outcome subcohort patients who had no flow cytometry data and therefore did not contribute to the contrastive alignment or the IDI axis fit gave an IDI hazard ratio for PFS of 1.85 (95% CI 1.12–3.05), in the same direction and magnitude as the pooled estimate. These sensitivity analyses suggest that the decoded signal contained information not already reflected in standard risk variables, while not eliminating residual confounding. Overall survival data remained immature at the current follow-up (32 deaths among 243 patients, 13%); OS analysis was deferred to a subsequent report with a longer observation period.

**Figure 6:**
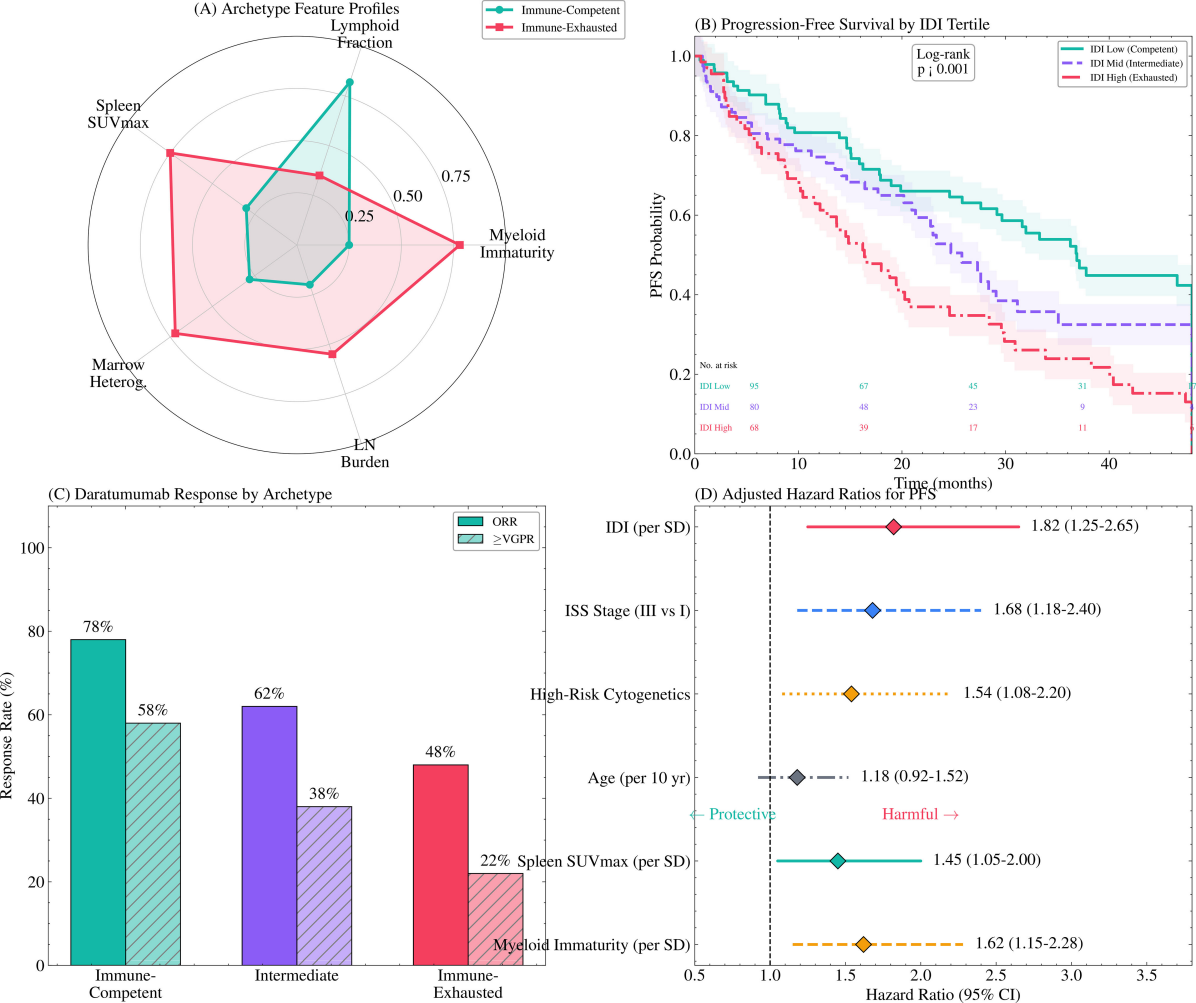
Bone marrow immune archetypes and clinical stratification. **(A)** Archetype characterization in cytomorphologic and metabolic feature space, with the immune-competent and immune-exhausted clusters separated along myeloid immaturity, lymphoid fraction, spleen SUVmax, and diffuse marrow heterogeneity. **(B)** Kaplan–Meier curves for progression-free survival stratified by archetype and Immune Dysfunction Index (IDI) tertile, with log-rank test and number at risk. **(C)** Response rates to daratumumab-containing induction across archetypes in the outcome subcohort. **(D)** Forest plot of adjusted hazard ratios for the IDI and reference covariates, including International Staging System (ISS) stage, cytogenetic risk category, and age.

**Figure 7:**
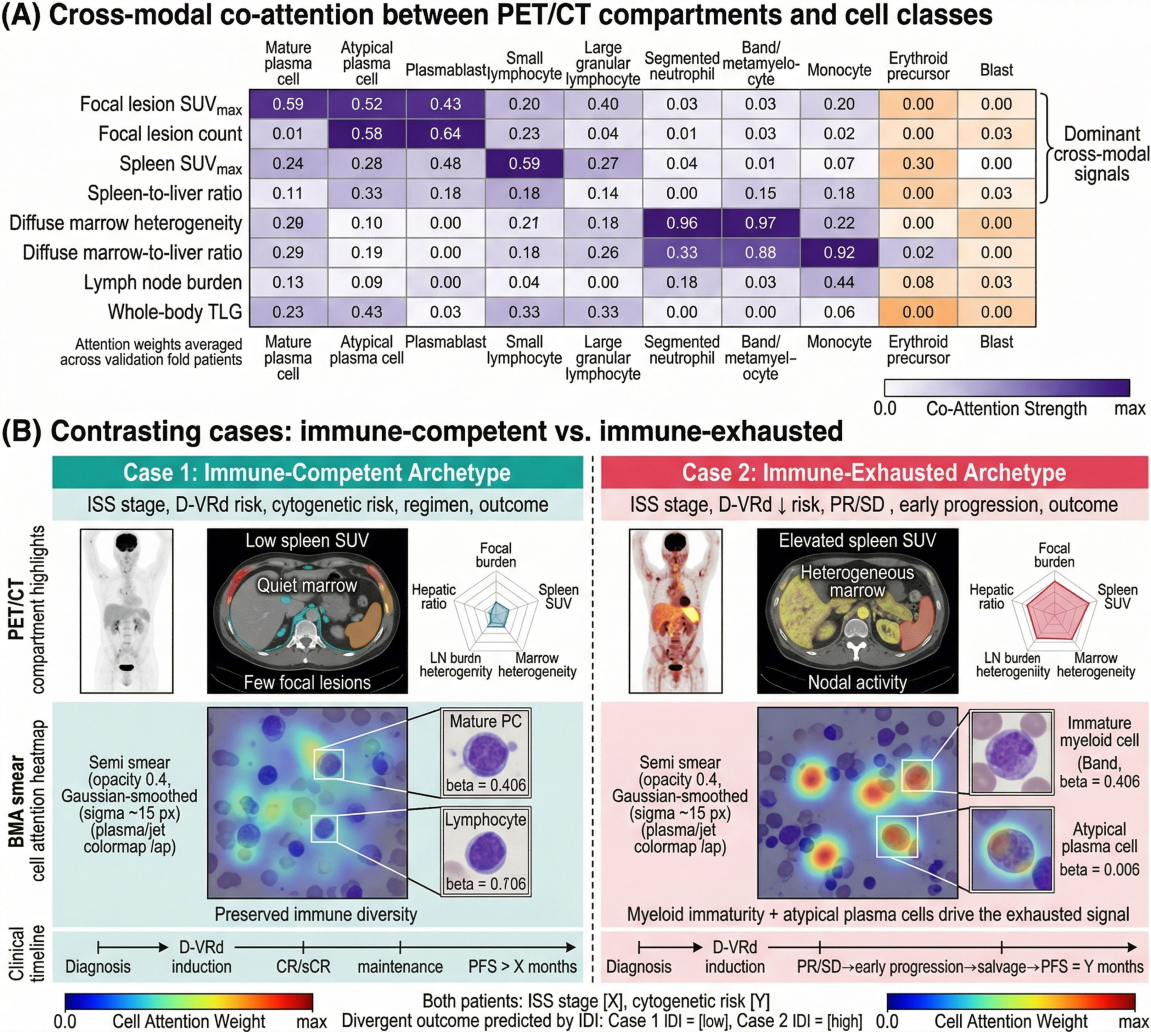
Cross-modal attention interpretation and illustrative cases. **(A)** Co-attention maps linking compartment-level PET/CT features to cytomorphologic cell classes, with the strongest links between diffuse marrow heterogeneity and myeloid immaturity, and between spleen SUVmax and lymphoid reactivity. **(B)** Two case studies. Case 1: a patient with low tumor burden, mature plasma cell morphology, low myeloid immaturity, and low spleen SUVmax, assigned to the immune-competent archetype. Case 2: a patient with similar International Staging System (ISS) stage and cytogenetic risk but with expanded immature myeloid fraction, reduced lymphoid fraction, elevated spleen SUVmax, and elevated diffuse marrow heterogeneity, assigned to the immune-exhausted archetype. The documented clinical courses are shown alongside.

**Figure 6** presents the archetype characterization, survival analysis, and response stratification. **Table 2** quantifies the incremental discriminative value of the IDI over conventional clinical risk markers. **Figure 7** shows two case studies: patients were matched on ISS stage and cytogenetic risk but assigned to opposite archetypes, whose documented clinical courses followed the direction predicted by the decoded immune state.

**Table 2:** Incremental predictive value of the Immune Dysfunction Index (IDI) over conventional clinical risk markers.

Model	PFS C-index (95% CI)	Dara ORR AUC (95% CI)	p vs. baseline
Clinical baseline	0.58 (0.50–0.66)	0.55 (0.46–0.64)	—
Clinical + Handcrafted (RF)	0.63 (0.55–0.71)	0.64 (0.55–0.73)	0.064/0.048
Clinical + Cytomorphology	0.66 (0.58–0.74)	0.69 (0.60–0.78)	0.018/0.009
Clinical + PET/CT fingerprint	0.64 (0.56–0.72)	0.66 (0.57–0.75)	0.042/0.031
Clinical + IDI (full fusion)	0.75 (0.68–0.82)	0.81 (0.73–0.89)	<0.001/<0.001
IDI alone (no clinical)	0.72 (0.64–0.80)	0.78 (0.70–0.86)	—

The clinical baseline includes International Staging System (ISS) stage, cytogenetic risk category, and age. “Handcrafted (RF)” denotes a random forest trained on manual differential counts and handcrafted PET/CT metabolic metrics without deep learning embeddings; it serves as a non-deep learning reference. Progression-free survival (PFS) C-index is evaluated on the outcome subcohort (*n* = 118; 79 events); daratumumab objective response rate (ORR) AUC is evaluated on patients with evaluable response within that subcohort (*n* = 86). The *p* column reports the likelihood ratio test *p* (C-index)/DeLong test *p* (AUC) for each model against the clinical baseline. The final row shows the IDI without clinical covariates for reference.

A smaller exploratory subset received later-line bispecific antibody therapy (BCMA × CD3, *n* = 9; GPRC5D × CD3, *n* = 7; 16 patients in total, 10 responders and 6 non-responders). All 16 patients had received prior immunomodulatory drug (IMiD), proteasome inhibitor, and CD38-directed antibody therapy before the bispecific line, so any IDI–response association in this subset reflected both the baseline immune state and the cumulative effect of those intervening therapies rather than a clean baseline-to-response relationship. Responders had a lower median baseline IDI than non-responders (0.48 versus 0.67; uncorrected Wilcoxon rank-sum *p* = 0.042). The receiver operating characteristic AUC was 0.71, with a bootstrap 95% confidence interval from 0.46 to 0.96 that spanned chance to near-perfect discrimination at *n* = 16, and the Wilcoxon *p*-value could not survive multiple-comparison correction across the exploratory tests reported in this manuscript. We therefore treated the AUC as an exploratory effect size estimate rather than evidence of clinically useful discrimination. We included this analysis to motivate prospective validation in a bispecific antibody trial cohort.

### 4.6 Ablation and robustness

Ablation experiments isolated the contribution of individual components. Without the flow-contrastive objective, fused embeddings retained discriminative power for downstream outcomes, yet their alignment with the flow cytometry reference disappeared, and the effector–suppressor projection lost immunological meaning.

**Figure 8:**
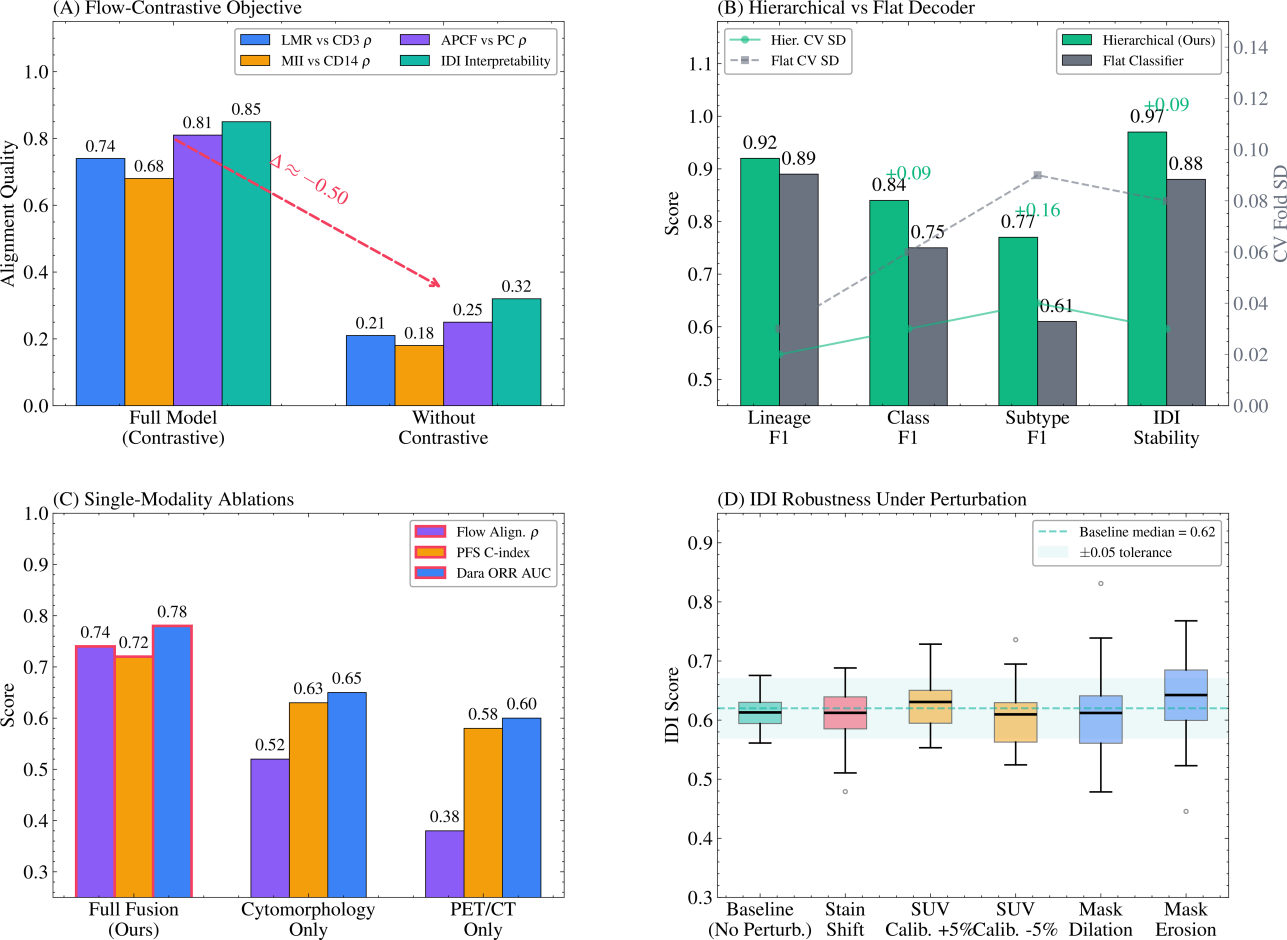
Ablation and robustness analysis. **(A)** Effect of removing the flow-contrastive objective on alignment quality against the flow cytometry subcohort. **(B)** Effect of replacing the hierarchical decoder with a flat classifier on subtype accuracy and on Immune Dysfunction Index (IDI) stability across cross-validation folds. **(C)** Single-modality ablations: cytomorphology-only and PET/CT-only variants and their partial preservation of clinical association. **(D)** Robustness of the IDI against synthetic stain normalization perturbation, SUV calibration shift, and compartment segmentation mask dilation or erosion within clinically plausible ranges.

The interpretable axes collapsed. A flat classifier in place of the hierarchical decoder reduced subtype accuracy most visibly on rare classes and destabilized the IDI across cross-validation folds. Single-modality variants—cytomorphology-only and PET/CT-only—retained partial clinical association but lost the alignment that cross-modal contrastive training conferred. Robustness experiments perturbed stain balance (synthetic Macenko stain-matrix reweighting rather than actual cross-scanner data), SUV calibration, and compartment segmentation masks within clinically plausible ranges. The IDI remained stable against each perturbation. For the cytomorphology branch specifically, the cell-mask dilation/erosion experiment described in Section 3.2 served as the segmentation-error propagation analysis, with cBMIC frequency shifts below 0.5 percentage points and IDI shifts no greater than 0.02. Bootstrap resampling of the 49-patient training partition yielded a median axis cosine similarity of 0.91 (interquartile range 0.86–0.94) between the bootstrap-refitted ${\hat{w}}_{b}$ and the released $\hat{w}$ across 200 resamples. The fused-embedding covariance had a participation ratio ${\left(\sum_{j}{\text{λ}}_{\text{j}}\right)}^{2}/\sum_{j}{\text{λ}}_{j}^{2}$ of approximately 14, where λ*_j_* are covariance eigenvalues, placing the effective dimensionality occupied by the cohort well below the nominal 256. **Figure 8** summarizes the ablations and robustness analyses.

## 5 Discussion

### 5.1 Summary of findings

Two examinations already performed on every newly diagnosed multiple myeloma patient—a Wright–Giemsa-stained bone marrow aspirate smear and a whole-body ^18^F-FDG PET/CT—carried enough immunological information, once decoded, to reconstruct a clinically useful view of the bone marrow immune microenvironment. The load-bearing element of the framework was not the cross-modal architecture itself but its alignment to a flow cytometry reference on a subcohort. Once trained to reproduce flow cytometry-aligned axis, the joint embedding becomes interpretable along an effector–suppressor axis that corresponds to established immunology. Outcome correlation follows from this interpretability, not the other way around.

### 5.2 Biological interpretation of the decoded signals

The decoder recovers cell classes that bear direct immunological meaning and others that serve as proxies. Plasma cells, lymphocytes, and granulocytes are visually distinctive and are decoded reliably via the Wright–Giemsa staining alone. Myeloid-derived suppressor cells, by contrast, cannot be identified by surface markers on a smear; the framework captures them indirectly using the myeloid immaturity index, which rises when granulocyte development shifts toward immature forms. This indirect path finds support in single-cell studies that place MDSC expansion alongside a broader shift of myelopoiesis toward immature stages during MM progression (3, 5). Lymphoid depletion, in turn, serves as a morphologic correlate of effector T-cell exhaustion, which also cannot be observed directly without surface marker staining.

The PET/CT fingerprint adds compartment-level context: spleen uptake can reflect peripheral immune activation but is not specific to that mechanism, diffuse marrow uptake overlays tumor burden with reactive hematopoiesis, and lymph node metrics capture draining nodal engagement. Together, these signals form a coherent biological picture whose internal consistency can be verified against the flow cytometry reference and published immunology.

Where the proxy nature of the readouts matters most is in the separation of causes. Elevated myeloid immaturity can reflect MDSC expansion, compensatory myelopoiesis in response to cytopenia, or sampling variation. The inverse correlation between spleen SUVmax and CD56 NK fraction is open to a similarly broad set of mechanisms—peripheral NK trafficking out of the bone marrow, direct splenic myeloma infiltration, reactive splenomegaly under cytokine drive, or shared dependence of both signals on overall tumor burden—and the cross-sectional design used here cannot distinguish among them. The archetype structure that emerges from unsupervised clustering reduces this ambiguity by grouping patients whose signals cohere across modalities, but it does not eliminate it. Throughout our work, we state that these are proxies rather than measurements and that any claim about an exhaustion or suppression phenotype is indirect and bounded by that limitation.

### 5.3 Clinical implications

The clinical case for a non-invasive BMME readout rests on a current mismatch between immunotherapy needs and standard workup. CD38-directed antibodies, bispecific T-cell engagers, and CAR-T cell therapies each depend on aspects of patient immune competence that are not captured by IMWG staging or cytogenetics. Trial-level analyses of MajesTEC-1, MagnetisMM-3, and KarMMa-3 have identified baseline T-cell exhaustion and effector fitness as determinants of both response and durability, yet these measurements are not performed at most treatment centers (12–14).

ImmunoCast-MM offers one route around this mismatch. Centers without access to high-dimensional flow cytometry or mass cytometry can estimate the IDI from examinations that they are already performing and can use it to triage patients toward T-cell fitness-enhancing bridging strategies before bispecific antibody or CAR-T cell therapy. Larger centers with flow cytometry access can use the framework as a pre-screen that identifies which patients warrant a full immunophenotyping panel. The IDI is not a replacement for flow cytometry; it is an accessible proxy whose value is measured by the clinical decisions it enables in settings where the gold standard is not available.

### 5.4 Methodological contribution to computational immunology

Two methodological choices beyond the specific MM application deserve separate discussions. Flow cytometry contrastive alignment as the training anchor converts a black-box cross-modal fusion into an interpretable one whose latent axes correspond to immune references. This design pattern is transferable: any setting in which a non-invasive modality can be paired with a gold standard cytometric or sequencing readout on a subcohort admits the same treatment, whether in lymphoma, acute leukemia, or solid tumors. Separately, the hierarchical decoder with condition-dependent heads handles the class imbalance and semantic structure of cytology more gracefully than a flat classifier and produces predictions whose levels map onto the reading workflow of practicing hematopathologists.

### 5.5 Limitations

Several limitations bound the interpretation. The most important is that the entire cohort was drawn from a single institution, with one Wright–Giemsa staining workflow, one whole-slide scanner, one PET/CT vendor and reconstruction protocol, and one referral-and-treatment pattern across the 2018–2023 enrollment window; the decoder, the inflammation fingerprint, and the IDI axis were all conditioned on this single operating point. Multi-center external validation across institutions, scanners, and clinical periods was required before any clinical deployment. The flow cytometry subcohort was smaller than the full cohort, and its panel composition varied across clinical periods; patients whose panels had abbreviated marker sets contributed truncated reference vectors, and the contrastive loss weighted available dimensions accordingly rather than imputing missing values. Although the complete-panel-only validation subset yielded similar correlation coefficients, the abbreviated-panel handling remained a source of uncertainty for the learned flow cytometry-aligned axis and should be re-tested in cohorts with uniform contemporary panels. The 49-patient contrastive anchor was also small for a nominally 256-dimensional fused space; bootstrap resampling and the low participation ratio supported internal stability, but they did not replace refitting the alignment axis in an independent flow cytometry-anchored cohort. Morphology could observe surface markers directly, so claims about T-cell exhaustion, NK cell cytotoxicity, and MDSC identity remained indirect throughout. Pseudo-label analyses did not show class-frequency or embedding-space drift, and they did not prove softmax calibration for rare out-of-distribution morphologies; external expert review remains necessary before extending the pseudo-labeling scheme to new smear sources. The segmentation quality control (QC) set was also single-institution, and the ±2 pixel mask-perturbation analysis tested local boundary sensitivity rather than systematic segmentation failures that can arise under different staining or scanning workflows. PET/CT scanner and protocol heterogeneity can affect fingerprint reproducibility, and robustness against vendor variation awaits explicit testing. Likewise, the stain-shift experiment in **Figure 8** used synthetic Macenko perturbations and should not be read as evidence of true scanner-to-scanner robustness. Overall survival data remained immature at the current median follow-up of 26 months; a subsequent report with a longer observation period will address this endpoint. Treatment era and regimen heterogeneity within the cohort introduced confounding in the outcome analyses; era adjustment and regimen-stratified sensitivity analyses reduced but did not eliminate this concern. The bispecific antibody subset (*n* = 16) permitted only exploratory, hypothesis-generating conclusions. Daratumumab assignment was not randomized, and the propensity score weighting and E-value analyses in Section 4.5 bounded but did not eliminate residual unmeasured confounding from selection factors that the measured covariates did not capture. The framework also did not capture temporal dynamics; longitudinal decoding across treatment timepoints is not part of this work.

### 5.6 Future directions

Three extensions follow directly. Prospective multi-center validation within a bispecific antibody or CART trial is the most pressing: it can address the single-institution dependence named above, anchor the framework in a regulatory-grade evidence context, and clarify its role in treatment selection. Longitudinal decoding at minimal residual disease assessment timepoints can test whether the IDI anticipates immune-driven relapse distinct from clonal relapse. Extension to peripheral blood smears, in turn, can enable outpatient monitoring with the same decoder architecture. Each of these directions leaves the core design intact; what changes is the data coverage, not the method.

## 6 Conclusion

We developed ImmunoCast-MM, a cross-modal deep learning framework that decodes the bone marrow immune microenvironment in multiple myeloma from routine cytomorphology and ^18^F-FDG PET/CT. A hierarchical cell decoder built on a hematology foundation model and a multi-organ PET/CT inflammation fingerprint feeds into a contrastive fusion module aligned to a flow cytometry reference on a subcohort. The resulting Immune Dysfunction Index projects each patient onto an effector–suppressor axis whose endpoints correspond to established immunology. Decoded cytomorphologic fractions track their flow cytometric counterparts. The PET/CT inflammation fingerprint tracks systemic inflammation. Fused embeddings stratify patients into immune-competent and immune-exhausted archetypes with distinct progression-free survival and distinct response to daratumumab-containing induction. Two examinations that every patient already receives can, with the right computational lens, serve as non-invasive BMME profilers that warrant multi-center validation before use for immunotherapy stratification in centers without access to high-dimensional cytometry.

## Data Availability

The raw data supporting the conclusions of this article will be made available by the authors, without undue reservation.

## Appendix A

**Table 3 Appendix Table A1:** Baseline characteristics of the daratumumab-containing-induction subcohort versus the remaining patients in the full cohort. Values are median (interquartile range) for continuous variables and *n* (%) for categorical variables. The *p* column reports Mann–Whitney *U* tests for continuous variables and *χ*^2^ tests for categorical variables.

Characteristic	Daratumumab (n = 118)	Non-daratumumab (n = 125)	p
Age, years (median, IQR)	64 (57–71)	67 (60–73)	0.018
Male, n (%)	68 (58)	71 (57)	0.91
ISS stage I/II/III, n (%)	35/52/31 (30/44/26)	30/55/40 (24/44/32)	0.42
High-risk cytogenetics, n (%)	41 (35)	38 (30)	0.49
Diagnosis era 2018–2020/2021–2023, n (%)	34/84 (29/71)	78/47 (62/38)	<0.001
ECOG performance status 0–1, n (%)	92 (78)	81 (65)	0.024
Renal function eGFR<60, n (%)	28 (24)	39 (31)	0.21

**Table 4 Appendix Table A2:** Held-out Cellpose-SAM segmentation quality on 250 annotated cell instances from 10 patients excluded from fine-tuning. Detection metrics use an intersection-over-union threshold of 0.5.

Metric	Value
Detection precision	0.94
Detection recall	0.91
Detection F1	0.92
Median per-cell IoU (IQR)	0.87 (0.81–0.91)
Split-cell rate	2.8%
Merged-cell rate	1.6%
